# Henri de Toulouse-Lautrec’s talent – and troubles – were larger than life

**DOI:** 10.1080/17571472.2017.1274936

**Published:** 2016-12-26

**Authors:** Francesco Carelli

**Affiliations:** Professor Family Medicine, Milan and Rome, Italy

[The Toulouse – Lautrec Collection of the Budapest Museum of Fine Arts at Ara Pacis, Rome, December 2015].

## The artistic story

Taking 170 works from the Museum of Arts in Budapest, Ara Pacis in Rome has produced a major exhibition on the painter-bohemian Henri Toulouse-Lautrec, of the late nineteenth century. It traces the artist’s life, and his rather premature death at only 36 years of age.

The exhibition, organized by Zetema, contains a broad spectrum of this artist’s work: posters, illustrations, sheets, music covers and posters. Some are rarities because they are printed in limited editions; signed, numbered and accompanied by the dedication of the artist.

The five sections of the exhibition cover the interests of the artist: the Parisian nightlife, with brothels and theaters of Montmartre; the actresses in vogue at the time, the famous can-can dancer La Gouloue, the horse races at Longchamp and new inventions.

The peculiarity of his art, unlike his contemporaries, is his using as a subject people, the proletariat and their entertainment. And this was utterly fascinating for the French *bourgeoisie*. They are representations of moments of everyday life that give effects to great immediacy. They earned him commissions for advertising, posters for plays, ballets and shows, as well as illustrations for magazines.

## The medical story

Toulouse-Lautrec’s short stature and brittle bones are indeed tempting fare for artists and scientists alike, but have medical historians ever really solved the mystery of what ailed the diminutive painter? Posthumous diagnoses have run from achondroplasia and other forms of dwarfism, to a combination of bad luck and syphilis.

During the mid-20th century, the commonly held thought was that Toulouse-Lautrec suffered from an inherited bone disease. At first, osteogenesis imperfecta was believed to be the most likely culprit, since the so-called brittle bone disease would explain why the painter’s bones fractured so easily when he was a young man. Others suggested the cause might be the rarer osteopetrosis, or marble bone disease, in which bones become dense and hard, prone to growth retardation and also fracture.

As understanding into another rare genetic disorder has grown, it seems that the debate has finally been settled. Pycnodysostosis seems the most likely culprit. Once the disease was identified by French physicians Pierre Maroteaux and Maurice Lamy in 1962, they declared an official end to the diagnostic debate on Toulouse-Lautrec.

Pycnodysostosis’s hallmark symptoms – virtually every one of which was displayed by Toulouse-Lautrec – include short stature, fragile bones, large skull, short distal phalanges, dental issues, a distinctive set of facial features including a weak chin and hooked nose, as well as open fontanelles of the sort that might be hidden under a bowler hat. His parental consanguinity may well clinch the diagnosis.

In 1996, the gene responsible for pycnodysostosis – now also known as Toulouse-Lautrec Syndrome – was found, making definitive diagnosis possible.

## The family story

As was common in many aristocratic French families, his parents, the Count Alphonse and the Countess Adèle de Toulouse-Lautrec, were first cousins and so their consanguinity was likely to have contributed to their young son’s congenital condition.

Henri wasn’t the only one to suffer it seems. At least three of his cousins, themselves the progeny, of cousins – also suffered from dwarfism and other congenital and skeletal defects. One relative was so fragile and tiny that she lived her entire life in a baby carriage.

Henri was fragile from birth. Short and sickly, he spent much of his childhood indoors watching other kids frolic about his family’s vast country estates. To add insult to injury, he was somewhat unattractive, he drooled, and he had a speech impediment with lifelong sinus troubles. When he was 13, Henri fractured his right femur; when he was 14, he fractured his left. His broken legs never properly healed, nor did they grow beyond that point, and so little Henri walked with a cane and a limp for the rest of his life.

The long months of recovery spent immobilized indoors, however, did contribute to his burgeoning love of art. Under the tutelage of his father, grandfather and uncle, themselves able draftsmen, as well as lessons from professional artists, Henri’s talents emerged.

By the time he was a young man, Henri had reached his full height of about 1.5 m. Though his torso and arms were average, his legs remained those of a child. His parents spared no expense in treating him, though nothing – not even electroshock therapies – could restore their son to good health or normal size.

Though somewhat possibly inspirational at first, absinthe dulled the pain of Toulouse-Lautrec’s social anxieties. He made sure he always had a supply of absinthe on hand by storing some in his hollow walking stick.

It was obvious to most who knew him that his medical situation was more than just a case of short stature. His facial features were quite distinctive, with a large forehead and nose, and a receding chin. He had terrible teeth and constant toothaches, too, and was rarely seen without that famous black hat, which most assumed he wore to add an extra inch or two of height. He admitted to friends that he suffered from syphilis as well, a situation that probably contributed to his declining mental health in later years.

Over time his alcoholism evolved into anxiety, depression, fear and paranoia. Hallucinations often took him over: friends one day found him shooting his pistol at invisible spiders. He believed the police were trying to arrest him and considered ill-meaning dogs were at every turn. In 1899, after making a very public scene in a brothel and experiencing a breakdown of sorts, Toulouse-Lautrec was dragged by his mother’s goons to a private insane asylum for three months.

He continued to take it easy for months after that, resting in the south of France, but he soon resumed his old habits, drinking heavily once again. A series of strokes resulting from alcoholism and tertiary syphilis began in March of 1901, resulting in greater paralysis. On 9 September 1901, a final stroke took his life. He died at 36, as famous for the scenes he painted as the scenes he made.

